# Dual locking plate fixation, PRP-augmented autologous bone grafting, and bioactive core construction for femoral fracture nonunion: a retrospective study of 52 cases

**DOI:** 10.3389/fmed.2025.1615628

**Published:** 2025-06-12

**Authors:** Zhihao Peng, Shiheng Wang, Ke Jie, Yonghong Dai, Kunyu Wang, Jiahua Wu, Feng Wu, Jianrong Chen

**Affiliations:** ^1^The Eighth Clinical Medical College of Guangzhou University of Chinese Medicine, Foshan, China; ^2^Foshan Hospital of Traditional Chinese Medicine, Foshan, China; ^3^Guangzhou University of Chinese Medicine, Guangzhou, China; ^4^Henan University of Chinese Medicine, Zhengzhou, China

**Keywords:** fracture nonunion, femoral fracture nonunion, platelet-rich plasma, autologous iliac bone grafting, dual plate fixation

## Abstract

**Background:**

Femoral nonunion remains a challenging orthopedic condition. This study evaluates a combined protocol integrating biomechanical stabilization (dual locking plate fixation) and maximal biological stimulation (PRP-augmented autologous bone grafting with bioactive core construction) to optimize bone healing.

**Methods:**

A retrospective analysis included 52 femoral nonunion patients treated at a tertiary trauma center (2020–2024). Outcomes assessed radiographic union (9-month and final follow-up), clinical union time, thigh incision healing, pain scores (VAS), lower extremity function (LEFS), and complications.

**Results:**

Cohort demographics: 35 males, 17 females; mean age 41.38 years, BMI 24.79 kg/m^2^. Nonunion subtypes: hypertrophic (36.5%, *n* = 19), atrophic (50%, *n* = 26), oligotrophic (13.5%, *n* = 7); locations: femoral shaft (63.5%, *n* = 33), supracondylar (36.5%, *n* = 19). All achieved union (mean follow-up: 19.01 months) with mean union time 6.56 ± 1.04 months. Postoperative outcomes: pain score 0.63 ± 0.97, LEFS 63.92 ± 5.92, incision healing 12.13 ± 1.36 days. The incidence rate of serious complications was 3.85% (2/52).

**Conclusion:**

The protocol demonstrated efficacy and safety, achieving rapid union (6.56 months), robust functional recovery (LEFS 63.92), and a low incidence of serious complications (3.85%). Biomechanical-biological integration represents a viable strategy for femoral nonunion management.

## Introduction

Fracture nonunion, defined by the U. S. FDA as failure to achieve union within 9 months or absence of radiographic healing progression over three consecutive months, is clinically characterized by persistent localized pain and pseudarthrosis (1). Femoral fracture nonunion specifically manifests as debilitating weight-bearing pain, potential abnormal mobility, and varying degrees of muscular atrophy, severely compromising lower limb function and imposing substantial burdens on patients’ quality of life, families, and society (2, 3).

Current strategies for managing femoral nonunion, as reported in the literature, are broadly categorized into three approaches: (1) Mechanical Stability Optimization: Biomechanical stability remains a prerequisite for healing (1, 4). Techniques include intramedullary nail dynamization, nail exchange, supplementary plate augmentation, combined nail-plate fixation, and external fixation with bone transport, with or without bone grafting. Despite these options, consensus on optimal fixation methods remains elusive (5–8). (2) Biological Augmentation: Enhancing osteogenic potential at the nonunion site is critical, particularly in atrophic cases (4, 9). Strategies include autologous bone grafting (ABG, considered the gold standard), bone morphogenetic proteins (BMPs), demineralized bone matrix (DBM), bone marrow mesenchymal stem cells (BMSCs), platelet-rich fibrin (PRF), and platelet-rich plasma (PRP). This strategy exhibits substantial innovative potential and has garnered significant interest among researchers, emerging as a focal theme in recent nonunion research. However, it currently lacks high-quality studies and robust supportive data (1, 4, 9), particularly regarding platelet-rich plasma (PRP) applications in fracture nonunion. Recent literature over the past 5 years predominantly consists of small case reports (*n* ≤ 5), underscoring the necessity for large-scale clinical studies to validate its efficacy (9, 10). (3) Non-invasive Interventions: Modalities such as electromagnetic field stimulation, extracorporeal shockwave therapy, ultrasound-guided percutaneous growth factor injection, and traditional Chinese medicine remain controversial in efficacy (1, 4). As an autologous blood product, PRP is enriched with bioactive factors such as platelet-derived growth factor (PDGF), transforming growth factor-beta (TGF-*β*), vascular endothelial growth factor (VEGF), epidermal growth factor (EGF), and insulin-like growth factor (IGF). It directly or indirectly activates the osteogenic differentiation of mesenchymal stem cells, promotes vascularization, optimizes the microenvironment, exerts anti-inflammatory and immunomodulatory effects, and enhances bone matrix synthesis and mineralization, thereby facilitating the bone tissue repair process (11–13).

Since January 2020, our team has employed dual plate fixation, PRP-augmented autologous grafting, and bioactive core construction for femoral nonunion, achieving favorable clinical outcomes. This study systematically evaluates the efficacy and safety of this biomechanically stable, biologically optimized strategy. Additionally, it aims to provide high-level evidence for PRP application in nonunion treatment and establish a reproducible protocol for PRP utilization in revision surgery.

## Patients and methods

This single-center retrospective case series, approved by the Ethics Committee of Foshan Hospital of Traditional Chinese Medicine (Approval No.: KY[2024]329), adhered strictly to the Declaration of Helsinki and ethical guidelines. Written informed consent was obtained from all participants. We reviewed cases of aseptic femoral fracture nonunion treated with revision surgery by a senior trauma team between April 2020 and June 2024. Diagnostic criteria followed the U. S. FDA definition: absence of radiographic union within 9 months or lack of healing progression over 3 months. Nonunion types were classified using the Weber-Cech system (hypertrophic, atrophic, oligotrophic). Exclusion criteria: skeletal immaturity, infected nonunion (confirmed histologically), initial open fractures, bone defects >5 cm, non-use of dual plate fixation, absence of PRP-augmented autologous iliac bone grafting (verified via surgical records), failure to construct a bioactive core (documented in records), or incomplete follow-up data. Patients with multiple fractures were excluded. Union criteria: radiographic evidence of ≥3 continuous cortical bone bridges on digital radiographs (DR) and absence of pain at the fracture site during full weight-bearing. Radiographic outcomes included union rates at 9 months post-revision and final follow-up. Clinical metrics encompassed time to union, thigh incision healing duration, pain scores at the nonunion site, lower extremity functional scores, and complications (defined as surgical site infection, incisional exudate/delayed healing, hematoma, heterotopic ossification, or thrombosis).

### Surgical procedure

The revision protocol for fracture nonunion strictly followed established methodologies (5, 14–16). All bone grafts were harvested from the anterior iliac crest, and revision surgeries at nonunion sites were performed through previous surgical incision scars.

**Figure 1:**
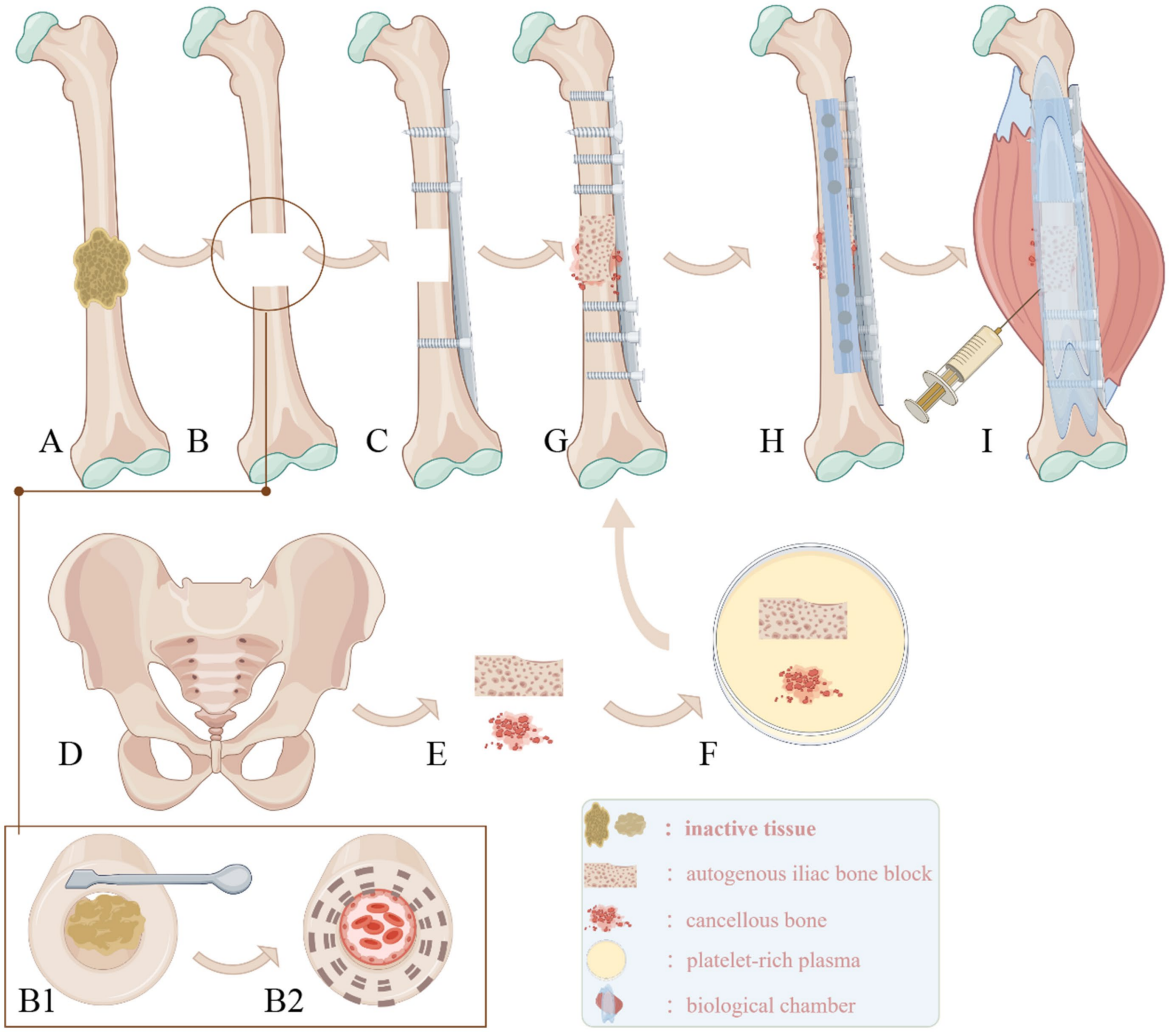
**(A)** Removal of failed internal fixation devices, with nonviable tissues surrounding the fracture ends. **(B1)** Debridement of nonviable tissues at the fracture site and recanalization of the medullary cavity, revealing the “red pepper sign.” **(B2)** Cross-sectional view showing fresh, bleeding bone surfaces and a patent, hemorrhagic medullary canal. **(C)** Mechanical axis correction followed by lateral locking plate placement. **(D,E)** Harvesting of a corticocancellous bone block from the anterior iliac crest, with additional cancellous bone chips collected. The bone block is trimmed to match the defect geometry. **(F)** Immersion of the graft in PRP solution. **(G)** Implantation of PRP-saturated bone grafts into the fracture site, with compression applied via the lateral locking plate and appropriate screw fixation. **(H)** Placement of an anterior auxiliary plate with compression. **(I)** Creation of a bioactive chamber by suturing periosteal and residual viable tissues around the fracture site, followed by injection of residual PRP into the chamber and its walls.

Step 1: Existing internal fixation devices were removed. Devitalized tissues (necrotic bone, sclerotic bone, and nonviable fibrous tissue) were meticulously debrided. The medullary canal was recanalized until bleeding bone surfaces (“red pepper sign”) were visualized. Excised bone specimens were submitted for histopathological analysis (Figures 1A,B1,B2, 2E).

**Figure 2:**
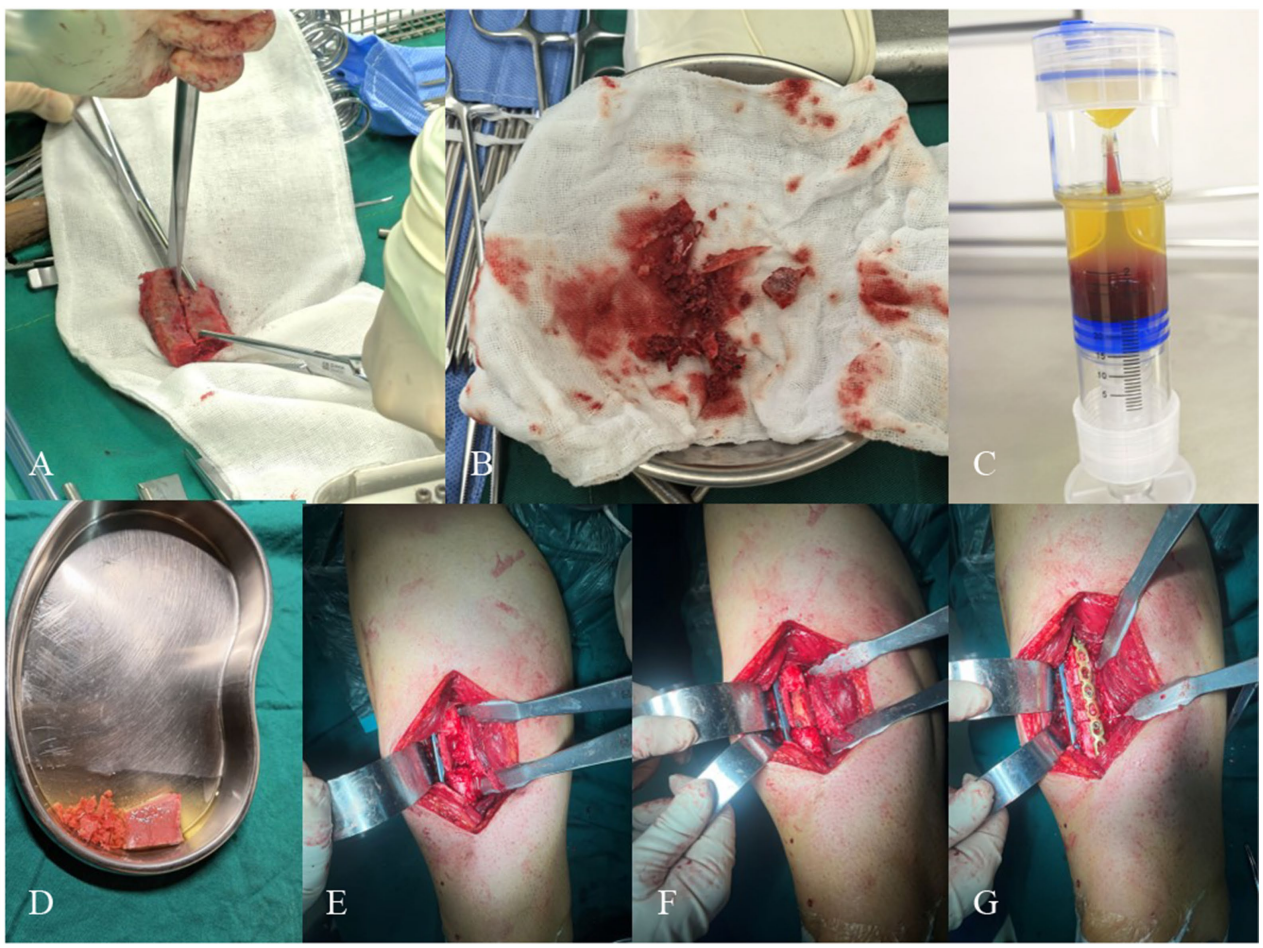
**(A,B)** A full-thickness corticocancellous bone block and cancellous bone chips are harvested from the anterior superior iliac spine region, then contoured to match the defect geometry. **(C)** Preparation of 10 mL platelet-rich plasma (PRP) at 4–5 × baseline platelet concentration. **(D)** Immersion of the shaped bone block in PRP. **(E)** Debridement of nonviable tissues to expose the “red pepper sign” at the fracture ends, followed by mechanical axis correction and deformity reduction. A lateral femoral locking plate is applied. **(F)** Implantation of the PRP-saturated corticocancellous block into the defect, with cancellous chips packed into residual gaps. **(G)** An anteromedial locking plate is positioned flush against the grafted bone and compressed.

Step 2: The preparation method of platelet-rich plasma strictly followed the methods reported in the literature (10, 11) and Chinese national standards. Forty-seven milliliters of blood was drawn from the elbow vein and mixed with 3 mL of sodium citrate solution for anticoagulation. The PRP tube was then sent to the laboratory and placed in a centrifuge, centrifuged at 2000 r/min for 10 min. After centrifugation, the PRP tube was taken out and allowed to stand for 5–10 min, finally obtaining 10 mL of platelet-rich plasma (Figure 2C). The prepared PRP was tested by laboratory counting to determine that the concentration was 4–5 times the original count (range: 700 × 10^9^/L ~ 900 × 10^9^/L).

Step 3: The surgical site was irrigated, followed by mechanical axis correction. A locking plate was positioned laterally on the femur, with appropriate screws inserted at both fracture ends to maintain reduction (Figures 1C, 2F).

Step 4: A full-thickness corticocancellous bone block, sized according to the defect dimensions, was harvested from the anterior iliac crest, along with particulate cancellous bone chips (Figures 1D,E, 2A,B).

Step 5: The bone block was trimmed to match the defect geometry. The shaped corticocancellous block and cancellous chips were immersed in 10 mL PRP solution for 5 min (Figures 1F, 2D).

Step 6: The PRP-saturated corticocancellous block was implanted into the defect, with cancellous chips densely packed into residual gaps. The lateral plate was compressed and secured with additional screws (Figures 1G, 2F).

Step 7: An anterior auxiliary plate was applied to compress the grafted bone block. Absorbable hemostatic gauze was placed for bleeding control (Figures 1H, 2G).

Step 8: Viable soft tissues surrounding the nonunion site were approximated using continuous absorbable sutures to create a sealed compartment. Residual PRP solution was injected into the “chamber walls” (Figure 1I).

Meticulous preservation of periosteal and soft tissue integrity was prioritized, with strict avoidance of vascular disruption. The surgical field was irrigated with 0.9% sodium chloride solution at 3 degrees Celsius to reduce bleeding, followed by placement and packing of absorbable hemostatic gauze, and sterile gauze packing with compression for hemostasis. During drill reaming, continuous irrigation with 0.9% sodium chloride solution at 3 degrees Celsius was applied at a flow rate of 1 mL per second to mitigate thermal injury. Tissue preservation at the nonunion site was maximized throughout the procedure.

### Postoperative rehabilitation and follow-up protocol

All patients received intravenous second-generation cephalosporin antibiotics intraoperatively through 48 h postoperatively for infection prophylaxis. Preoperative counseling included lifelong smoking cessation. Daily aseptic dressing changes were performed until incision healing, with delayed healing defined as wound closure exceeding 14 days (documented via outpatient records). Bedside ankle pumps and quadriceps strengthening exercises commenced on postoperative day 1. Within 2–3 weeks postoperatively, partial weight-bearing walking training is performed using a walker. Subsequently, weight-bearing is gradually increased according to clinical and imaging improvements until full weight-bearing is achieved.

Follow-ups occurred at 1, 2, 3, 6, 9, and 12 months postoperatively, then biannually or as needed. Radiographic union was evaluated via X-rays at each visit; CT scans supplemented ambiguous cases. Imaging interpretations were independently verified by a senior orthopedic trauma specialist and two radiologists blinded to the study. Pain assessment and functional scoring (lower extremity functional scale) were conducted at the first confirmed union by a senior orthopedic surgery graduate student unaffiliated with the research. All participants provided written consent for data utilization under institutional ethics committee oversight.

### Statistical analysis

Data were processed using SPSS (v26.0; IBM Corp., Armonk, NY). As a single-group case series analysis, intergroup comparative statistics were not performed.

## Results

**Figure 3:**
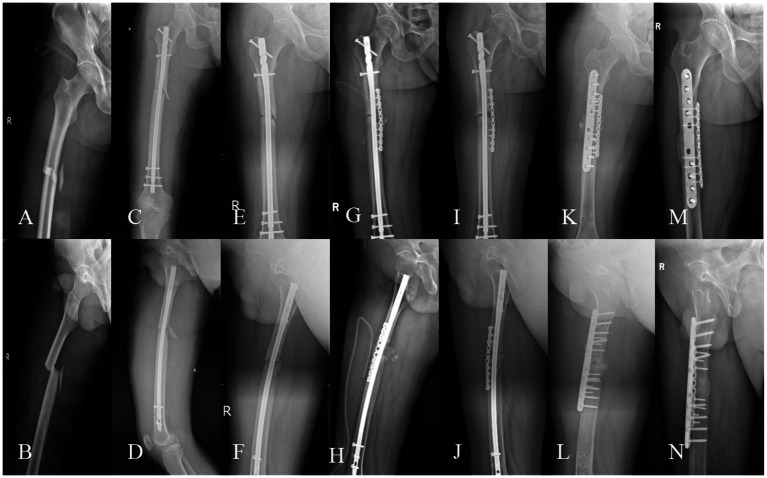
A 39-year-old male, BMI: 22.16 kg/m^2^, non-smoker, no metabolic diseases. **(A,B)** Right femoral shaft fracture sustained in a car accident on January 5, 2021. **(C,D)** Closed reduction and intramedullary nailing performed on January 13, 2021. **(E,F)** Nonunion with minimal callus formation observed during follow-up on October 8, 2021. **(G,H)** Revision surgery on October 23, 2021, retaining the original nail and augmenting with autologous iliac cancellous bone grafting and an auxiliary plate. **(I,J)** Persistent nonunion noted during follow-up on July 7, 2022. **(K,L)** Second revision surgery on July 12, 2022, utilizing dual locking plate fixation, PRP-augmented structural iliac bone grafting, and bioactive core construction. **(M,N)** Excellent radiographic union observed at 6.2 months postoperatively, with a lower extremity functional score of 65 and a VAS pain score of 1.

**Figure 4:**
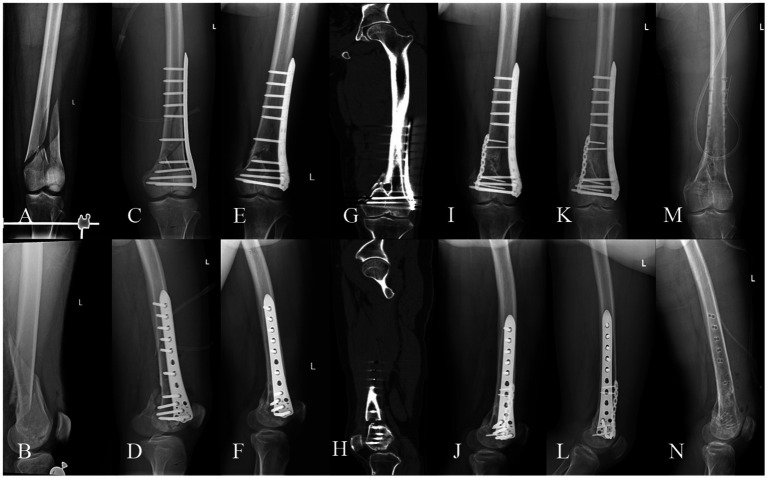
A 37-year-old male, BMI: 25.98 kg/m^2^, 2-year smoking history, no metabolic diseases. **(A,B)** Left distal femoral comminuted fracture sustained in a car accident on January 19, 2022. **(C,D)** Open reduction and lateral locking plate fixation performed on January 29, 2022. **(E,F)** Delayed union observed during follow-up on December 23, 2022. **(G,H)** Nonunion with anterior-medial cortical defect confirmed by CT on June 6, 2023. **(I,J)** Revision surgery on July 21, 2023, retaining the lateral locking plate and augmenting with an anterior-medial auxiliary plate, PRP-augmented structural iliac bone grafting, and bioactive core construction. **(K,L)** Radiographic union confirmed at 6.4 months postoperatively (February 2, 2024), with a lower extremity functional score of 60 and a VAS pain score of 0. **(M,N)** Implant removal performed on March 19, 2024.

From 98 initially reviewed medical records, 45 cases were excluded: 25 lacking dual plate fixation, 7 without PRP application, 5 without autologous iliac grafting, 5 infected nonunions, 2 segmental bone defects, and 1 incomplete documentation. Of the 53 included patients, one died in a traffic accident at 5.2 months postoperatively, leaving 52 final participants (35 males, 17 females; mean age 41.38 ± 13.95 years, range 18–69). Nonunion classifications included hypertrophic (19, 36.5%), atrophic (26, 50%), and oligotrophic (7, 13.5%), with 33 femoral shaft (63.5%) and 19 supracondylar (36.5%) cases. All patients completed follow-up (mean 19.01 ± 6.69 months, range 12–45). Baseline characteristics—including interval between initial and revision surgery, injury mechanism, anatomical location, and prior fixation methods—are detailed in Table 1. All 52 patients achieved union (Table 2), with a mean time to clinical and radiographic union of 6.56 ± 1.04 months (range 5–10.3). At 9-month follow-up, 50 patients (96.2%) met union criteria; all achieved union by final follow-up (100%). Representative cases are shown in Figures 3, 4. At initial union confirmation, mean visual analog scale (VAS) pain score was 0.63 ± 0.97 (range 0–5), lower extremity functional score (LEFS) averaged 63.92 ± 5.92 (range 52–76), and thigh incision healing time was 12.13 ± 1.36 days (range 10–16). Postoperatively, 5 patients (9.62%) had complications: 2 donor site discomforts after exercise, 1 femoral vein thrombosis, 1 chronic knee pain (VAS 5), and 1 delayed thigh incision healing (healed day 16). No hematoma or heterotopic ossification occurred. Two patients (3.85%) had serious complications: 1 femoral vein thrombosis (resolved with 1 month oral rivaroxaban) and 1 chronic knee pain (improved with oral diclofenac and topical flurbiprofen gel).

**Table 1:** Demographic information and baseline characteristics of 52 patients.

Variable	Result
Gender	
Female	17 (32.69%)
Male	35 (67.31%)
Age(years)	41.38 ± 13.95 (18–69)
BMI(kg/m^2^)	24.79 ± 1.53 (22.14–28.24)
Number of surgeries	1.54 ± 0.70 (1–3)
Time from the first fracture surgery to the revision surgery (months)	15.81 ± 8.89 (5–48)
Nonunion area	
Femoral shaft	33 (63.5%)
Supracondylar part of femur	19 (36.5%)
Type of nonunion	
Hypertrophic	19 (36.5%)
Atrophic	26 (50%)
Oligotrophic	7 (13.5%)
Injury mechanism	
Traffic accident	28 (53.8%)
Fall	24 (46.2%)
Location	
Left	24 (46.2%)
Right	28 (53.8%)
Preoperative fixation method	
Intramedullary nail	31 (59.6%)
Plate	21 (40.4%)
VAS before revision surgery	4.54 ± 1.02 (3–8)
LEFS before revision surgery	24.67 ± 5.91 (11–39)

Categorical data are presented in the form of quantity and percentage; continuous data are presented in the form of mean ± standard deviation and range. VAS, Visual Analogue Scale; LEFS, Lower Extremity Functional Scale.

**Table 2:** Clinical and imaging outcomes of 52 patients.

Variable	Result
Healing status in the ninth month after revision surgery	
Healed	50 (96.2%)
Not healed	2 (3.8%)
Healing status at the last follow-up	
Healed	52 (100%)
Not healed	0 (0%)
Average healing duration (months)	6.56 ± 1.04 (5–10.3)
Length of hospital stay (days)	11.92 ± 4.95 (5–32)
VAS after revision surgery	0.63 ± 0.97 (0–5)
LEFS after revision surgery	63.92 ± 5.92 (52–76)
Healing duration of the thigh incision (days)	12.13 ± 1.36 (10–16)
Follow-up duration (months)	19.01 ± 6.69 (12–45)
Blood loss (milliliters)	477.88 ± 370.53 (100–1,500)
Surgery duration (minutes)	186.56 ± 54.77 (62–294)
Complications	5 (9.62%)

Categorical data are presented in the form of quantity and percentage; continuous data are presented in the form of mean ± standard deviation and range. VAS, Visual Analogue Scale; LEFS, Lower Extremity Functional Scale.

## Discussion

Femoral fracture nonunion, a complication with profound functional implications (2, 3), lacks consensus on optimal management despite numerous reported strategies, with conflicting success rates across methods (5–8). Our protocol—combining dual locking plate fixation for biomechanical stability, PRP-augmented autologous iliac bone grafting, and bioactive core construction—achieved 100% union rates, accelerated healing (mean 6.56 months), excellent pain control (mean VAS 0.63), and functional recovery (mean LEFS 63.92), enabling early rehabilitation. Representative cases are shown in Figures 3, 4.

**Figure 5:**
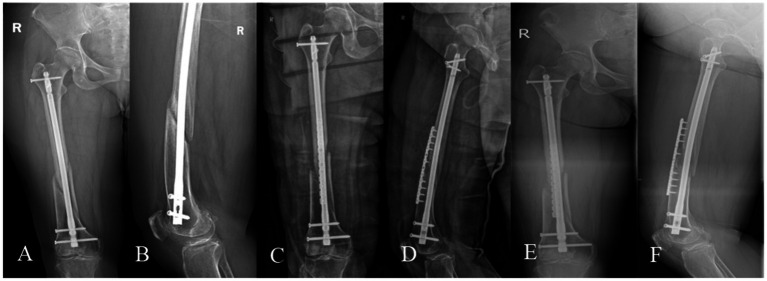
A 57-year-old female, BMI: 26.35 kg/m^2^, sustained a right femoral shaft fracture from a 2-meter fall on March 2, 2024, treated elsewhere with closed reduction and intramedullary nailing. **(A,B)** Nonunion with minimal callus formation observed during evaluation at our institution on December 15, 2024. **(C,D)** Revision surgery on December 26, 2024, retaining the original nail and augmenting with an anterior auxiliary plate and PRP-augmented iliac bone grafting. **(E,F)** Screw cutout at the proximal end of the anterior plate observed during follow-up on February 28, 2025.

Current literature reports several internal fixation methods for aseptic femoral fracture nonunion, including intramedullary nail dynamization, nail exchange, combined nail-plate fixation, and dual plating techniques (5–8, 14). Among these, nail exchange has gained considerable favor among researchers (17). Upsizing the intramedullary nail enhances resistance to axial bending forces, improving mechanical stability, while reaming-generated intramedullary cancellous debris and blood flow optimize the local biological environment. This approach is recognized for concurrently offering “mechanical benefits” and “biological benefits” (18, 19). Another common strategy involves augmenting retained or exchanged intramedullary nails with supplementary plates, with or without bone grafting. This nail-plate hybrid construct addresses rotational stability deficiencies inherent to standalone nails. Biomechanical studies (20, 21) demonstrate that hybrid systems exhibit 3.3-fold greater bending and torsional resistance compared to isolated nails. Clinical evidence (19, 22) confirms higher success rates (95–100%) for nail-plate constructs versus nail exchange alone (82.8–91.7%). Despite reported successes with intramedullary nailing, this nail-centric strategy demonstrates limited advantages in treating non-isthmal fractures, extensive bone defects, and atrophic nonunions. Bone defects exceeding 5 mm have been identified as a risk factor for failure of nail exchange (18, 23). Intramedullary nail fixation does not allow sufficient bone grafting, which limits its efficacy in atrophic nonunions (18, 19, 24). This limitation has been corroborated by Özkan et al. (17). Studies also indicate that reaming to accommodate larger-diameter nails damages endosteal vasculature, thereby impairing biological healing processes (25). Placing plates over intramedullary nails makes it challenging to insert adequate bicortical screws (22, 25). Monocortical fixation, due to reduced screw working length, increases the risk of screw cutout and subsequent fixation failure (Figure 5). Intramedullary nailing struggles to achieve satisfactory reduction in cases of malalignment or multiple fracture fragments at the nonunion site (24, 26). The difficulty and failure rate of intramedullary nailing increase when addressing broken screws or fractured nails from prior fixation (27). Additionally, intramedullary nailing is inappropriate for metaphyseal-diaphyseal nonunions and elevates the risk of failure (18). Studies further note that if the initially implanted nail is already of maximum diameter, no larger nail can be provided during revision surgery (14, 28). These limitations highlight the necessity for alternative fixation strategies in complex scenarios, particularly those requiring simultaneous biological optimization and robust mechanical control.

Biomechanical stability is a critical determinant of healing success (1, 4). Biomechanical studies demonstrate that compression plates provide superior tension band structures compared to intramedullary nails (14), with dual plating offering enhanced rotational stability, particularly in non-isthmal fractures (5, 8, 22). Our protocol—dual locking plate fixation combined with structural iliac bone grafting—effectively optimizes biomechanical stability. In a 54-case study (14), authors achieved 100% union rates (mean healing time: 8.23 months) using locking compression plates with autografts (iliac and vascularized fibular bone) for femoral and tibial nonunions. However, their reliance on vascularized fibular grafts increased surgical complexity and required extended remodeling time. In contrast, our technique achieved shorter healing durations with simplified surgical execution. Chamseddine et al. (29) reported successful nonunion management using bridging plates with bone grafting but emphasized aggressive periosteal decortication. Our approach minimized excessive periosteal stripping, reducing associated risks. Wang et al. (5), in a 186-case femoral nonunion series, utilized dual plating with a 4 cm × 1 cm bone trough for graft placement, achieving 98.4% union rates (mean healing time: 7.6 months). While effective, their method involved significant bone sacrifice, which remains controversial in atrophic nonunion management (30). Comparatively, our protocol reduced bone loss and accelerated healing (6.56 months). Similarly, Taheriazam et al. (8) achieved 100% union rates via medullary iliac grafting and dual plating but reported limb shortening in 29.55% of cases. Our technique eliminated this risk. Gavaskar et al. (31) treated 38 cases of distal femoral nonunion with a combination of lateral locking plates, medial anatomical locking plates, and autologous iliac bone grafting, achieving a 91% union rate. The medial anatomical plates addressed the difficulties in distal positioning of the medial condyle and insufficient screw working length caused by non-anatomical implants, but their placement induced knee joint dysfunction and pain, requiring secondary surgery for complication management in 24% of patients. In the study by Lotzien et al. (32), a large augmenting plate was implanted via a medial approach after debridement and bone grafting while retaining the original lateral locking plate. Although a satisfactory union rate was achieved, this technique was associated with substantial surgical trauma and irritation of the medial thigh muscles and soft tissues by the medial plate. In contrast to the above techniques, this study uses anterior-medial placement of a small auxiliary plate without additional surgical incisions, causing less trauma to the medial thigh tissues, improving patient comfort, and facilitating limb functional recovery. These studies collectively affirm that rigid fixation is pivotal for femoral nonunion healing (5, 8, 14, 29, 31, 32). In our series, dual locking plates established a low-strain mechanical environment, optimizing conditions for biological consolidation.

Biological stimulation holds equal importance to biomechanical stability. While biological agents have demonstrated success in nonunion management, robust evidence supporting their superiority over autologous bone grafting—particularly autologous iliac crest bone grafting (AICBG), the current gold standard for atrophic nonunions (1, 4, 9)—remains lacking (9). Reliance solely on ABG may yield inconsistent outcomes (33), prompting clinicians to combine biological agents with ABG. A recent review by the American Academy of Orthopedic Surgeons highlights a 95% success rate with multimodal therapies (30). Commonly used biologics include bone morphogenetic proteins, demineralized bone matrix, and platelet-rich plasma (4, 9). Though FDA-approved for nonunion treatment, BMPs lack conclusive evidence of outperforming AICBG (4, 9, 42). Recent studies increasingly combine BMPs with ABG for fracture nonunion treatment (4, 9). A latest 24-case prospective series (34) reported 100% union rates using rhBMP-2, hydroxyapatite, and autografts for long bone nonunions, suggesting benefits of BMP-AICBG (autologous iliac crest bone grafting) combinations. However, concerns persist regarding BMP-2 antibody-induced adverse immune responses and exuberant bone formation. Despite BMPs’ promise, their application remains contentious (35), with reported drawbacks including high costs and complications such as inflammatory reactions, hematoma, radiculopathy, heterotopic ossification, osteoclast activation, osteolysis, and tumorigenesis (36, 37). A 20-year-old randomized controlled trial (RCT) of 120 cases (38) compared rhBMP-7 and PRP for long bone nonunions, demonstrating superior outcomes with rhBMP-7 (union rate: 86.7% vs. 68.3%; healing time: 8 vs. 9 months). The absence of ABG in the PRP cohort likely contributed to inferior efficacy, as PRP monotherapy underperforms combination approaches (9, 10). Additionally, suboptimal PRP concentrations in that study may have impaired results. Most scholars posit that PRP at 3–6 × baseline platelet levels optimally promotes cellular proliferation, migration, and collagen synthesis, while exceeding 6 × may suppress osteoblast activity (10–12). In our protocol, PRP at 4–5 × baseline concentration enhanced AICBG, yielding safe and superior outcomes. PRP, an autologous blood product, contains concentrated platelets and growth factors—including platelet-derived growth factor (PDGF-aa, PDGF-bb, PDGF-ab), transforming growth factor (TGF-β1, TGF-β2), vascular endothelial growth factor (VEGF), and epidermal growth factor (EGF). These mediators facilitate bone repair and regeneration by stimulating mesenchymal stem cell migration, proliferation, and osteogenic differentiation (11–13).

Choi et al. (34) demonstrated that hydroxyapatite granules and the microporous structure of autologous cancellous bone effectively load BMP, enabling sustained growth factor release. They also constructed a soft tissue template around the nonunion site to guide anatomically conforming bone formation while preventing BMP leakage, termed “conformed bone formation.” Similarly, Nie et al. (16) achieved 93.75% union rates by packing defects with a “putty-like” composite of demineralized bone matrix (DBM) powder mixed with PRP, protected via soft tissue coverage to optimize the regenerative microenvironment. In our protocol, PRP-saturated autologous iliac crest bone grafts utilized cancellous bone’s microporosity (Figure 1F; Figure 2D) to retain substantial PRP volumes, facilitating prolonged bioactive release and enhancing the “bone healing unit’s” potency. Chamseddine et al. (29) achieved 100% union without soft tissue complications using periosteal decortication and tight soft tissue closure to maximize graft-host contact. Our technique sutured viable periosteal and fascial tissues around the nonunion, creating a sealed compartment (Figure 1I) that replicated the “biological chamber” described by Calori and Giannoudis (15), establishing a “maximal bioactive core” central to the diamond concept. PRP within this chamber exerted anti-inflammatory effects. A prospective RCT (39) comparing intra-articular PRP versus saline in pilon fractures reported significantly lower IL-6, IL-8, and PGE2 levels with PRP, alongside elevated IL-1RA, mitigating post-traumatic arthritis risk. Recent studies (40) corroborate concentrated platelet plasma’s ability to accelerate wound healing via fibroblast and endothelial cell migration/proliferation, aligning with Zhang et al.’s (41) findings. Nie et al. (16) further observed reduced incisional complications in PRP cohorts, underscoring its clinical value in inflammation modulation and tissue repair. These advancements underscore the synergistic potential of biomechanical stabilization and targeted biological augmentation in overcoming traditional therapeutic limitations.

In our study, the dual locking plate fixation system established a stable biomechanical environment, while PRP-augmented autologous iliac bone grafting combined with bioactive core construction achieved maximal biological stimulation, accelerating early bone healing and remodeling. This approach enabled earlier functional rehabilitation, potentially reducing complication risks by shortening disability duration (DVT incidence: 1.92%). Functional outcomes, including a mean Lower Extremity Functional Scale (LEFS) score of 63.92 and a pain score of 0.63, confirmed significant quality-of-life improvements.

Notably, current literature on PRP-augmented grafting for femoral nonunion remains scarce (4, 9, 10), precluding direct comparative analysis. As a retrospective study, potential biases—despite mitigation through blinded radiographic assessments (senior orthopedic specialist + two independent radiologists) and functional evaluations by non-affiliated researchers—include selection and assessment biases. Additional limitations include the absence of a control group, lack of nonunion subtype stratification, exclusion of large bone defects (exceeding 5 cm), and variability in PRP preparation protocols. Future research should prioritize biomechanical comparisons between nail-plate and dual plating systems to validate mechanical advantages. Additionally, multicenter randomized trials are needed to define the indications and efficacy of PRP-grafting protocols, especially for large bone defects when combined with ABG. These efforts will refine the stepwise therapeutic framework for traumatic nonunion.

## Conclusion

This study validates a safe and effective protocol for femoral nonunion by integrating dual locking plate fixation (providing biomechanical stability) with PRP-augmented autologous iliac grafting and bioactive core construction (enabling growth factor gradient release). The strategy achieved a mean union time of 6.56 months (range: 5–10.3 months), a 3.85% serious complication rate (DVT: 1.92%), and marked functional recovery (mean LEFS: 63.92; pain score: 0.63). Critically, the synergy between mechanical stabilization and biological stimulation not only accelerated healing but also reduced secondary complications by minimizing disability duration, establishing a novel paradigm for restoring limb functional integrity.

## Data Availability

The raw data supporting the conclusions of this article will be made available by the authors, without undue reservation.

## References

[1] Wildemann B Ignatius A Leung F Taitsman LA Smith RM Pesántez R. Non-union bone fractures. Nat Rev Dis Primers. (2021) 7:57. doi: 10.1038/s41572-021-00289-8, PMID: 34354083

[2] Wagner RK Emmelot MP Ly TV Harris MB Janssen SJ Kloen P. Long-term patient reported outcomes after revision surgery for lower extremity nonunion: a retrospective cohort study. Injury. (2024) 55:111779. doi: 10.1016/j.injury.2024.111779, PMID: 39146614

[3] Nicholson JA Makaram N Simpson A Keating JF. Fracture nonunion in long bones: a literature review of risk factors and surgical management. Injury. (2020) 52:S3–S11. doi: 10.1016/j.injury.2020.11.029 33221036

[4] Siverino C Metsemakers WJ Sutter R Della Bella E Morgenstern M Barcik J. Clinical management and innovation in fracture non-union. Expert Opin Biol Ther. (2024) 24:973–91. doi: 10.1080/14712598.2024.2391491, PMID: 39126182

[5] Wang C Sun L Wang Q Ma T Zhang K Li Z. The technique of “autologous bone grafting through channels” combined with double-plate fixation is effective treatment of femoral nonunion. Int Orthop. (2022) 46:2385–91. doi: 10.1007/s00264-022-05519-6, PMID: 35849163

[6] Ebraheim NA Martin A Sochacki KR Liu J. Nonunion of distal femoral fractures: a systematic review. Orthop Surg. (2013) 5:46–50. doi: 10.1111/os.12017, PMID: 23420747 PMC6583155

[7] Tsang ST Mills LA Baren J Frantzias J Keating JF Simpson AH. Exchange nailing for femoral diaphyseal fracture non-unions: risk factors for failure. Injury. (2015) 46:2404–9. doi: 10.1016/j.injury.2015.09.027, PMID: 26489394

[8] Taheriazam A Mir Ahmadi A Abbaszadeh A Soleimani M Darabi R Samberani M. Double plating and iliac crest bone graft can safely fix femoral shaft nonunion. Sci Rep. (2024) 14:28988. doi: 10.1038/s41598-024-79513-w, PMID: 39578535 PMC11584805

[9] Gagnon D Mouallem M Leduc S Rouleau DM Chapleau J. A systematic scoping review of the latest data on orthobiologics in the surgical treatment of non-union. Orthop Traumatol Surg Res. (2024) 110:103896. doi: 10.1016/j.otsr.2024.103896, PMID: 38663743

[10] Andersen C Wragg NM Shariatzadeh M Wilson SL. The use of platelet-rich plasma (PRP) for the management of non-union fractures. Curr Osteoporos Rep. (2021) 19:1–14. doi: 10.1007/s11914-020-00643-x, PMID: 33393012 PMC7935731

[11] Giusti I Rughetti A D’Ascenzo S Millimaggi D Pavan A Dell’Orso L. Identification of an optimal concentration of platelet gel for promoting angiogenesis in human endothelial cells. Transfusion. (2008) 49:771–8. doi: 10.1111/j.1537-2995.2008.02033.x 19170984

[12] Van Lieshout EMM Den Hartog D. Effect of platelet-rich plasma on fracture healing. Injury. (2020) 52:S58–66. doi: 10.1016/j.injury.2020.12.005 33431160

[13] Roffi A Di Matteo B Krishnakumar GS Kon E Filardo G. Platelet-rich plasma for the treatment of bone defects: from pre-clinical rational to evidence in the clinical practice. A systematic review. Int Orthop. (2016) 41:221–37. doi: 10.1007/s00264-016-3342-9, PMID: 27888295

[14] Ding P Chen Q Zhang C Yao C. Revision with locking compression plate by compression technique for Diaphyseal nonunions of the femur and the tibia: a retrospective study of 54 cases. Biomed Res Int. (2021) 2021:9905067. doi: 10.1155/2021/9905067, PMID: 34368357 PMC8346318

[15] Calori GM Giannoudis PV. Enhancement of fracture healing with the diamond concept: the role of the biological chamber. Injury. (2011) 42:1191–3. doi: 10.1016/j.injury.2011.04.016, PMID: 21596376

[16] Nie W Wang Z Cao J Wang W Guo Y Zhang C. Preliminary outcomes of the combination of demineralized bone matrix and platelet rich plasma in the treatment of long bone non-unions. BMC Musculoskelet Disord. (2021) 22:951. doi: 10.1186/s12891-021-04840-2, PMID: 34781964 PMC8594103

[17] Özkan S Nolte PA van den Bekerom MPJ Bloemers FW. Diagnosis and management of long-bone nonunions: a nationwide survey. Eur J Trauma Emerg Surg. (2018) 45:3–11. doi: 10.1007/s00068-018-0905-z, PMID: 29335752 PMC6394533

[18] Brinker MR O’Connor DP. Exchange nailing of ununited fractures. J Bone Joint Surg Am. (2007) 89:177–88. doi: 10.2106/JBJS.F.00742, PMID: 17200326

[19] Kook I Oh CW Shon OJ Kim JW Kim JW Hwang KT. Comparing outcomes of plate augmentation, nail exchange, and nail exchange with plate augmentation in the treatment of atrophic femoral shaft nonunion after intramedullary nailing: a multicenter retrospective study. Arch Orthop Trauma Surg. (2024) 144:1259–68. doi: 10.1007/s00402-023-05183-4, PMID: 38372763

[20] Park K Kim K Choi YS. Comparison of mechani-cal rigidity between plate augmentation leaving the nail in situand interlocking nail using cadaveric fracture model of thefemur. Int Orthop. (2011) 35:581–5. doi: 10.1007/s00264-010-0983-y, PMID: 20213515 PMC3066311

[21] Walcher MG Day RE Gesslein M Bail HJ Kuster MS. Augmentative plating versus exchange intramedullary nailing for the treatment of aseptic non-unions of the femoral shaft-a biomechanical study in a SawboneTM model. J Pers Med. (2023) 13:650. doi: 10.3390/jpm13040650, PMID: 37109036 PMC10142865

[22] Devendra A Patra SK Velmurugesan P Zackariya M Ramesh P Arun Kamal C. Results of a simple treatment protocol for aseptic femoral shaft nonunion in 330 patients. Injury. (2024) 55:111412. doi: 10.1016/j.injury.2024.111412, PMID: 38341997

[23] Tsang ST Mills LA Frantzias J Baren JP Keating JF Simpson AH. Exchange nailing for nonunion of diaphyseal fractures of the tibia: our results and an analysis of the risk factors for failure. Bone Joint J. (2016) 98-B:534–41. doi: 10.1302/0301-620X.98B4.34870, PMID: 27037437

[24] Shih CY Kor CT Hsieh CP Chen CL Lo YC. Does nail size or difference between canal and nail diameter influence likelihood of union or time to union of femoral shaft fractures treated with intramedullary nailing? A retrospective cohort study. BMC Musculoskelet Disord. (2022) 23:826. doi: 10.1186/s12891-022-05781-0, PMID: 36045444 PMC9429295

[25] Uliana CS Bidolegui F Kojima K Giordano V. Augmentation plating leaving the nail in situ is an excellent option for treating femoral shaft nonunion after IM nailing: a multicentre study. Eur J Trauma Emerg Surg. (2020) 47:1895–901. doi: 10.1007/s00068-020-01333-0, PMID: 32107562

[26] Lin SJ Chen CL Peng KT Hsu WH. Effect of fragmentary displacement and morphology in the treatment of comminuted femoral shaft fractures with an intramedullary nail. Injury. (2013) 45:752–6. doi: 10.1016/j.injury.2013.10.015, PMID: 24268188

[27] Wu T Zhang W Chang Z Zhu Z Sun L Tang P. Augmented stability in leaving original internal fixation with multidimensional cross locking plate through mini-open femoral anterior approach for aseptic femoral shaft nonunion: a retrospective cohort study. Orthop Surg. (2022) 15:169–78. doi: 10.1111/os.13581, PMID: 36411511 PMC9837237

[28] Nadkarni B Srivastav S Mittal V Agarwal S. Use of locking compression plates for long bone nonunions without removing existing intramedullary nail: review of literature and our experience. J Trauma. (2008) 65:482–6. doi: 10.1097/TA.0b013e31817c9905, PMID: 18695487

[29] Chamseddine AH Mouchantaf ME Freiha KF Asfour AH Dib AA Wardani HM. Bridge plating with decortication, autologous bone graft, and tight closure: a “stepwise surgical diamond concept” for treatment of nonunion in a series of fifty five patients. Int Orthop. (2022) 46:1241–51. doi: 10.1007/s00264-022-05379-0, PMID: 35306570

[30] Wellings EP Moran SL Tande AJ Hidden KA. Approach to tibial shaft nonunions: diagnosis and management. J Am Acad Orthop Surg. (2024) 32:237–46. doi: 10.5435/JAAOS-D-23-00453, PMID: 38190574

[31] Gavaskar AS Tummala NC Reddy CR Gopalan H Srinivasan P. What is the likelihood of union and frequency of complications after parallel plating and supplemental bone grafting for resistant distal femoral nonunions? Clin Orthop Relat Res. (2023) 482:362–72. doi: 10.1097/CORR.0000000000002809, PMID: 37638842 PMC10776157

[32] Lotzien S Baron D Rosteius T Cibura C Ull C Schildhauer TA. Medial augmentation plating of aseptic distal femoral nonunions. BMC Musculoskelet Disord. (2023) 24:554. doi: 10.1186/s12891-023-06675-5, PMID: 37407946 PMC10324211

[33] Schmidt AH. Autologous bone graft: is it still the gold standard? Injury. (2021) 52:S18–22. doi: 10.1016/j.injury.2021.01.043, PMID: 33563416

[34] Choi W Kim BS Cho WT Lim EJ Choi JS Ryu YK. Efficacy and safety of recombinant human bone morphogenetic protein-2 (rhBMP-2) combined with autologous bone for the treatment of long bone nonunion: a report of a prospective case series. Injury. (2024) 55:111711. doi: 10.1016/j.injury.2024.111711, PMID: 39003882

[35] Carragee EJ Hurwitz EL Weiner BK. A critical review of recombinant human bone morphogenetic protein-2 trials in spinal surgery: emerging safety concerns and lessons learned. Spine J. (2011) 11:471–91. doi: 10.1016/j.spinee.2011.04.023, PMID: 21729796

[36] Fuchs T Stolberg-Stolberg J Michel PA Garcia P Amler S Wähnert D. Effect of bone morphogenetic protein-2 in the treatment of long bone non-unions. J Clin Med. (2021) 10:4597. doi: 10.3390/jcm10194597, PMID: 34640615 PMC8509770

[37] James AW LaChaud G Shen J Asatrian G Nguyen V Zhang X. A review of the clinical side effects of bone morphogenetic protein-2. Tissue Eng Part B Rev. (2016) 22:284–97. doi: 10.1089/ten.TEB.2015.0357, PMID: 26857241 PMC4964756

[38] Calori GM Tagliabue L Gala L d’Imporzano M Peretti G Albisetti W. Application of rhBMP-7 and platelet-rich plasma in the treatment of long bone non-unions: a prospective randomised clinical study on 120 patients. Injury. (2008) 39:1391–402. doi: 10.1016/j.injury.2008.08.011, PMID: 19027898

[39] Zitsch BP James CR Crist BD Stoker AM Della Rocca GJ Cook JL. A prospective randomized double-blind clinical trial to assess the effects of leukocyte-reduced platelet-rich plasma on pro-inflammatory, degradative, and anabolic biomarkers after closed pilon fractures. J Orthop Res. (2021) 40:925–32. doi: 10.1002/jor.25123, PMID: 34185333

[40] Fan L Zhang Y Yin X Chen S Wu P Huyan T. The effect of platelet fibrin plasma (PFP) on postoperative refractory wounds: physiologically concentrated platelet plasma in wound repair. Tissue Eng Regen Med. (2024) 21:1255–67. doi: 10.1007/s13770-024-00665-x, PMID: 39400879 PMC11589050

[41] Zhang S Tan H Cheng X Dou X Fang H Zhang C. Autologous platelet-rich fibrin enhances skin wound healing in a feline trauma model. BMC Vet Res. (2024) 20:504. doi: 10.1186/s12917-024-04358-4, PMID: 39508248 PMC11539556

[42] Xie C Wang C Huang W Huang Y Li Q Yu C. Recombinant human bone morphogenetic protein is a valid alternative to autologous bone graft for long bone non-unions: a systematic review and meta-analysis. Surgeon. (2023) 21:e173–82. doi: 10.1016/j.surge.2022.11.004, PMID: 36682906

